# Cultural repertoires and food-related household technology within *colonia* households under conditions of material hardship

**DOI:** 10.1186/1475-9276-11-25

**Published:** 2012-05-15

**Authors:** Wesley R Dean, Joseph R Sharkey, Cassandra M Johnson, Julie St John

**Affiliations:** 1 Program for Research in Nutrition and Health Disparities, Department of Health Promotion & Community Health Sciences, School of Rural Public Health, TAMU 1266, College Station, TX, USA, 77843; 2 Center for Health Promotion and Disease Prevention, The University of North Carolina at Chapel Hill, 1700 Martin Luther King Blvd, CB# 7426, Chapel Hill, NC, 27599-7426, USA; 3 Department of Nutrition, Gillings School of Global Public Health, The University of North Carolina at Chapel Hill, 260 Rosenau Hall, CB #7461, Chapel Hill, NC, 27599-7461, USA; 4 Center for Community Health Development, Texas A&M School of Rural Public Health, TAMU 1266, College Station, TX, USA, 77843

## Abstract

**Introduction:**

Mexican-origin women in the U.S. living in *colonias* (new-destination Mexican-immigrant communities) along the Texas-Mexico border suffer from a high incidence of food insecurity and diet-related chronic disease. Understanding environmental factors that influence food-related behaviors among this population will be important to improving the well-being of *colonia* households. This article focuses on cultural repertoires that enable food choice and the everyday uses of technology in food-related practice by Mexican-immigrant women in *colonia* households under conditions of material hardship. Findings are presented within a conceptual framework informed by concepts drawn from sociological accounts of technology, food choice, culture, and material hardship.

**Methods:**

Field notes were provided by teams of *promotora-*researchers (indigenous community health workers) and public-health professionals trained as participant observers. They conducted observations on three separate occasions (two half-days during the week and one weekend day) within eight family residences located in *colonias* near the towns of Alton and San Carlos, Texas. English observations were coded inductively and early observations stressed the importance of technology and material hardship in food-related behavior. These observations were further explored and coded using the qualitative data package Atlas.ti.

**Results:**

Technology included kitchen implements used in standard and adapted configurations and household infrastructure. Residents employed tools across a range of food-related activities identified as forms of food acquisition, storage, preparation, serving, feeding and eating, cleaning, and waste processing. Material hardships included the quality, quantity, acceptability, and uncertainty dimensions of food insecurity, and insufficient consumption of housing, clothing and medical care. Cultural repertoires for coping with material hardship included reliance on inexpensive staple foods and dishes, and conventional and innovative technological practices. These repertoires expressed the creative agency of women *colonia* residents. Food-related practices were constrained by climate, animal and insect pests, women’s gender roles, limitations in neighborhood and household infrastructure, and economic and material resources.

**Conclusions:**

This research points to the importance of socioeconomic and structural factors such as gender roles, economic poverty and material hardship as constraints on food choice and food-related behavior. In turn, it emphasizes the innovative practices employed by women residents of colonias to prepare meals under these constraints.

## Introduction

The *colonias* of the South Texas border region are largely Spanish-speaking neighborhoods composed of Mexican-immigrant and Mexican-origin U.S. residents with limited access to healthy foods [1], high levels of food insecurity [2], low health related quality of life [3], and greater than national and state level prevalence of adult diabetes [4] and obesity [4]. With the importance of daily food-related behaviors to dietary health outcomes in mind, *promotora-*researchers (indigenous community health workers) [5] were asked to observe and participate in the daily household activities of women residing in South Texas *colonias*. It was discovered that food-related behaviors were shaped by their limited material resources: the built environment, including the material infrastructure of homes and neighborhoods; the tools used in daily food-related activities; and their sometimes limited food supplies. In turn, women residents creatively employed a repertoire of inexpensive staple foods and dishes; inexpensive, common, and mostly unspecialized household items; and home infrastructure to accommodate the socioeconomic, biological and physical constraints of their environment, especially the material hardships endemic to life in the *colonias*.

## Background

### The *Colonia* home-environment

The population of *colonias* along the Texas border with Mexico is almost entirely Hispanic, and the household income of approximately 80% of the residents are at or below the poverty level [6]. *Colonias* are unincorporated functionally-rural communities built on subdivided land. *Colonias* serve as an archetypal example of new-destination Mexican immigrant communities [7-10]. *Colonias* typically consist of low-income housing, non-existent to inadequate infrastructure, and self-build dwellings [5]. In 2006, among the six largest Texan counties with *colonias*, there were 1,092 *colonias* with a population of 249,675 residents with full or partial services and 442 *colonias* with a population of 62,675 residents without services [11]. The *colonias* in this study were located in Hidalgo County which is home to more than 70% of Texas border colonias, suffers from persistent poverty and is one of the ten poorest counties in the United States [12]. In 2010, the population of Hidalgo County was 89.8% Hispanic or Latino, and 34.8% of families were below the poverty level [13].

*Colonia* homes have been described as self-build [6] housing. Self-build housing is provisional and improvisational. The owner implements the majority of planning and construction with assistance from friends and family. Construction often begins with a trailer (e.g. travel or pop-up trailer, recreational vehicle, or mobile home), or building a small residence. Residents expand and improve their homes as capital becomes available. The layout of homes is continually in flux and the purposes of spaces within the home change through time.

A common characteristic of *colonias* is their limited access to basic infrastructure. The absence or limited presence of potable water, drainage, sewers, gas, and electricity informs this understanding of the hardships experienced by *colonia* households [6,14]. The presence of services such as power, water, sewer and gas which would allow the *colonia* to function like a standard U.S. incorporated neighborhood or subdivision is limited due to endemic neighborhood poverty and the extra-jurisdictional location of many *colonias*[6]. Likewise, the integration of homes within *colonias* depends on whether they are connected directly to services [6].

### Food choice in the material environment of the *Colonia* household

**Figure 1:**
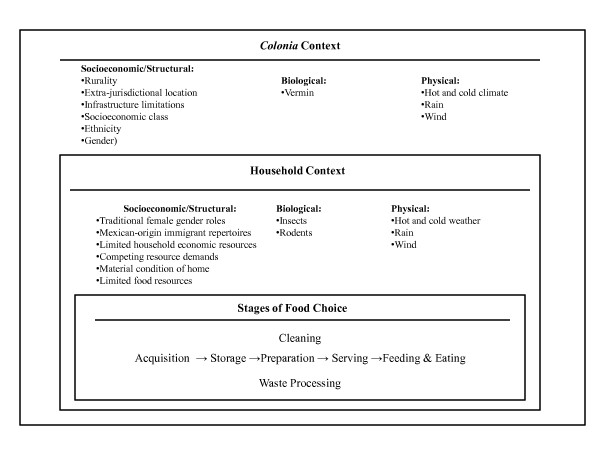
**Conceptual Model.** The contextual setting of food-related technological behaviors.

Food choice has been described by Sobal and colleagues as operating within a nested system of contexts [15]. In Figure 1, the structural contexts that frame food choice among *colonia* households are described. These include the household setting, which is situated at the intersection of the household with biological, physical and socioeconomic contexts [15]. The household is further nested within a neighborhood described in Figure 1 as the *colonia* context.

Food choice entails a material context which includes food-related technology. Research on household technology emphasizes the importance of examining the effect of that item’s use as it interacts with other elements of the home food environment, and with intervening human and non-human forces from outside the home [16-23].

The socioeconomic contexts that frame the use of food-related household technology are rooted in an account of culture that examines the interplay between human agency and the structural constraints that arise from membership in heterogeneous social group formations such as race and ethnicity, gender and socioeconomic class [24-29]. Contemporary sociological accounts of culture describe it as a repertoire or toolkit [25] an actor can employ to evaluate and devise solutions to problems such as food-related tasks [25,30-32]. The cultural repertoires that pervade one’s life as a member of a specific social group do not determine one’s actions. Rather, they simultaneously constrain and enable social action [24,26,27]. Tool use, much as any other form of social action [16,19,33], is framed by the strictures and possibilities of one’s cultural repertoire [25].

In this paper it is argued that food choices are acts of agency enabled by cultural repertoires. The selection of particular foods or ingredients, the preparation of particular dishes, or the use of food-related technology, are in part the product of the constraints and opportunities of an individual’s social position as defined within the constraints of social structure. Within the *colonia* setting, the employment of cultural repertoires such as food-related technological practices are largely constrained rather than enabled by social structure. The term material hardship employed by scholars of poverty to describe the condition of households that fall below minimum standards for the consumption of basic needs is used to capture these constraints on agency [34,35].

In this study, the agents of food choice express cultural repertoires to cope with conditions of material hardship. The forms of material hardship provide a structural context that frames the cultural repertoires used by Mexican-origin women residents of these *colonia* households. These cultural repertoires were in turn evidenced in their selection of foods and dishes, and in their food-related technological practices.

## Methods

### Data collection

In this participant-observation project, households were observed by two bilingual teams of trained researchers, consisting of a local *promotora-*researcher and a researcher with a graduate degree in public health. Each team conducted two four-hour weekday visits and one eight-hour weekend visit to four households for a total of eight households between the two teams. The *promotoras* in this study were previously involved in delivering services to these colonias. They recruited households based on prior familiarity, and their expectation the participants would be likely to complete the study. This research was approved by the Texas A&M University Institutional Review Board and study participants signed an informed consent form.

Participant observers were provided with an observational guide to help describe and understand the food choice and eating patterns of *colonia* residents. Participant observers were instructed to note the material aspects of the home food environment including where food was obtained; what foods were preferred by families; how food affected family interactions; what happened during food preparation (including where food was prepared and by whom) and the main techniques of preparation; and to describe characteristics of the specific family. In this study, participant observation allowed access to tacit technological practices that would be difficult to discover without direct observation [36-38]. Further detail on data collection and the study population is available elsewhere [39].

### Analysis

One stream of coding identified household technology and its multiple uses. Standard uses and user adaptations were identified for each item. In a second stream of coding, the factors that interact with users and technological items were identified and categorized. A third coding stream involved the deductive identification of food insecurity status and other material hardships. The process followed a technique established and validated by Hamelin and colleagues [40] to determine a household’s food insecurity status based on interview data. This technique relies on a criterion for food insecurity established by Frongillo and colleagues where one asks “would a reasonable person conclude that the household was insecure, considering the generally accepted definition of food insecurity (certainty, acceptability, quality and quantity of food) [41]?” Observational records were coded to identify poor food quality, limited food quantity, and problems with social acceptability and uncertainty over food resources. Observations were also coded to identify components of material hardship including problems with housing and crowding, inadequate clothing, health problems, and limited access to medical care [34].

## Results and discussion

### Environmental setting

Table 1 describes common household indicators of material hardship including the certainty, acceptability, quality and quantity dimensions of food insecurity. All eight households in the study exhibited some form of material hardship and only one did not exhibit any of the four dimensions of food insecurity. Two families described occasions where adults experienced anxiety and uncertainty about being able to find sufficient food to feed their families. Six families described the quality dimension of food insecurity. Specifically, they described regular meals consisting of a few staples including rice, beans, potatoes, and eggs, and inexpensive dishes such as soups and instant noodles. They also described their preference for the rare occasions where meat is featured, especially grilling meat outdoors with their extended family. Five families described the quantity dimension of food insecurity, specifically running out of food and having insufficient money and formal food assistance to obtain more. Four families reported the acceptability dimension of food insecurity when they ran out of money and benefits. These families sought food from church charities, family members, friends and neighbors.

**Table 1:** Families classified by indicators of material hardship

**Family**	**Food Insecurity**	**Housing**	**Residents/ Rooms**	**Clothing**	**Health**	**Health Care Access**
1	uncertainty, quality, quantity, acceptability	jacked, lighting, heat, insulation, hot-water, structure	7/4	clothing, used	m, f	no insurance, health expense
2	uncertainty, quality, quantity, acceptability	heat, insulation, hot-water	7/3	used	f	
3		lighting, heat, insulation, hot-water	7/3		m, f	
4	quality, quantity, acceptability	lighting, heat, insulation,	6/3		m	no insurance, health expense
5	quality	jacked, lighting, heat, insulation, hot-water	4/1			
6	quality	lighting, heat, insulation, hot-water, structure	3/1			health expense
7	quality, quantity, acceptability	heat, insulation, hot-water, structure	7/3			
8	quantity	heat, insulation,	7/3	used	m	

*Residents to rooms* is the ratio of residents to bedrooms and living room, not including separate kitchens; *uncertainty* indicates participants were uncertain they would be able to feed their family; *quality* indicates nearly exclusive consumption of inexpensive staple foods and limited consumption of preferred items such as fresh fruits, vegetables and meat; *quantity* indicates the family described situations where they ran out of food and did not have money or federal assistance to obtain more food; *acceptability* indicates the family had to rely on potentially unsustainable resources to obtain food when they ran out of money and formal food assistance such as church charities, borrowing from neighbors and relatives, and buying on credit; *jacked* indicates extension cord connection to electrical grid via neighbor; *lighting* indicates inadequate lighting such as candles, table lamps and broken or burned out light bulbs; *heat* indicates no central heating; *insulation* indicates no insulation in the home; *hot-water* indicates no working hot-water heater; *structure* indicates inadequate housing such as broken windows, doors, holes in flooring or leaks in roof and ceiling; *clothing* indicates insufficient funds to purchase adequate clothing; *used* indicates used clothing; *m* indicates poor adult male health; *f* indicates poor adult female health; *no insurance* indicates no health insurance, *health expense* indicates the family cannot afford medical care.

Categories of social group formation were associated with constraining and enabling the actions of women as they selected ingredients and dishes, and used food-related technology. These constraints and opportunities included conventional gender roles as home centered caretakers of children rather than wage earners, and the selection of ingredients and dishes common among Mexican-origin immigrants to the U.S. Distinct forms of material hardship were also identified: limited household financial resources and the built-environment at the household level, and limited neighborhood infrastructure and systemic poverty at the *colonia* level. Food-related behavior was also influenced by biological and physical characteristics of the *colonia* setting such as vermin, wind, rain, heat, and cold weather.

The mothers in this study were entirely of Mexican national origin, although many of the children were born in the U.S. All of the families described frequent interactions with family members still in Mexico, and many family members were currently dealing with the U.S. immigration process. Mexican-immigrant cultural repertoires were expressed through the dietary choices, culinary techniques, and tools used to prepare food. Foods and food practices were largely indicative of traditional Mexican foodways, although families had incorporated new foods widespread throughout both the U.S. and Mexico.

The observers recorded what they described as “typical Mexican food,” including regionally popular and traditional dishes such as *fideo* (pasta cooked in a chile inflected tomato broth), *menudo* (tripe and hominy stew), *charro* beans (bean soup), *tamalestacosmole*, and *tortillas*[42]. Foods not identified as traditionally Mexican but which are common in both Mexican and Mexican-American diets included a variety of American soft drinks, oatmeal, sugar-sweetened cereals, orange juice, peanut-butter and jelly sandwiches, and luncheon-meat sandwiches. Techniques familiar to the Mexican and Mexican-American home [42-47] included the use of blenders and *molcajetes* (mortar and pestle) to create soup bases from garlic, spices, and chiles; the charring and grilling of vegetables for salsas and soups; and tortilla making with a variety of presses.

The foods most commonly prepared in these households demonstrate the interrelationship of constraint and opportunity present in the interplay between cultural repertoire and material hardship. The many inexpensive foods typically identified as common in Mexican-origin households present in these households represent a cultural repertoire. The six families that indicated the quality dimension of food insecurity relied heavily on the least expensive of these staple items. These ingredients included rice, beans, *fideo* pasta, hotdogs, nixtamalized corn flour, wheat flour, and small quantities of low-quality ground meat. While better cuts of meat were mentioned, regionally popular meats and meat dishes were largely described as luxury items that were only featured on festive occasions. Meat and fish were not commonly consumed at home. In these households, socioeconomic status restricted regular opportunities to prepare desirable meat dishes and constrained the choice of ingredients and dishes to inexpensive staple items. Nevertheless, a cultural repertoire that took the form of dishes based on these inexpensive staples, allowed women for the most part to feed their families under conditions of material hardship. Another cultural repertoire was a fasting and feasting dynamic where regular meals were composed of inexpensive staples and bimonthly or monthly extended family feasts where meats were grilled outdoors on charcoal or wood fires. This repertoire allowed households the occasional opportunity to enjoy luxury foods.

Traditional gender roles were another constraint that shaped food-related technical practices within *colonia* homes. As observed elsewhere by Abarca [45] and Johnson and colleagues [48] the kitchen was set aside for the Mexican-American women in this study, and household labor was delineated according to traditional gendered expectations for low-income Mexican-immigrant families [49]. A public/private distinction shaped technical practices within the households. All the men were employed to some extent outside the home, and all the women in this study worked within the home and not for wages with the exception of one woman who took on occasional cleaning duties in nearby homes. Household food-related practices were largely but not entirely the work of women. In turn, women were the principal innovators who adapted kitchen technologies to the conditions of *colonia* life. These gendered cultural forms were made apparent through the performance of specific food-related technological tasks described in greater detail in the following section.

There were three categories of material hardship that shaped food-related technology use in *colonia* households. At the household level, limited household resources curtailed residents’ capacity to acquire foods and household items, especially well-made, expensive, and specialized items. Income poverty also had an effect on the construction and condition of homes. The homes in this study were representative of self-build housing. Many were formed around a core trailer unit with additions. Construction of additions was also ongoing in a number of the homes.

Household indicators of material hardship are detailed in Table 1. Five families were observed with inadequate lighting. Some homes used light bulbs in some, rather than all kitchen bulb sockets, thus reducing the money spent on bulbs and electricity. Some families also used candles during times of severe hardship. Homes also lacked built-in lighting-solutions. In these cases, portable lighting such as desk lamps lighted food preparation and eating spaces. Two homes without a direct connection to the electrical grid relied on what Ward has referred to as jacked electricity accessed through an electrical extension to their neighbor’s home and an electrical bill shared with the neighbor [6]. None of the homes had central heating, air conditioning, or insulated walls. Six homes were without functioning hot-water heaters, and three of the homes had serious structural inadequacies including broken windows, dangerous holes in the flooring and serious leaks and water damage. All of the households were crowded with a ratio of 2.3 residents per each bedroom or living room, not including separate kitchens or bathrooms. A number of other unmet household expenses included the three families observed wearing inadequate or used clothing, the five families with chronic health problems, and the three who said they did not have health insurance or who had health expenses they could not afford to pay. At the *colonia* level, systemic material hardship often takes the form of limited availability of utilities within *colonias*. However, the *colonias* in this study had available water, sewer, drainage, and electrical services. Food related systemic hardship was expressed by the limited spatial access of all households to grocery stores and supermarkets, complicated by the limited availability of public transportation.

### Kitchen technology in the household food environment

An organizational structure was developed to classify the uses of food-related technology. The complete list of items observed in these *colonia* homes is too lengthy to be reproduced here. The stages of this organizational structure are shown in Figure 1. They include acquisition, storage, preparation, feeding, and eating, cleaning, and waste processing. Acquisition, preparation, feeding, and eating correspond to DeVault’s stages of food choice [50] and the input and output categories of Sobal and colleagues [15]. Storage facilitates the continued availability and edibility of food [15]. Feeding is a transitional stage separated from eating because distinct actors may be feeding or eating [30]. Feeding also involves a distinct set of technical practices from preparation or eating, although these practices may be performed with the same tool. Eating captures embodied tool use, and the importance of the availability of adequate space and furniture in the home to the act of consumption. This includes tools used for eating such as utensils, and the tables, chairs and other items used to support the body during the act of consumption. Cleaning and waste processing are separated from the process depicted in Figure 1 because they are associated with all the above stages [15]. Cleaning and waste processing can both take place as foods are prepared for consumption or as one cleans up after cooking or eating a meal.

*Acquisition* of food involved a number of distinct techniques. Although no gardening was reported, two families raised chickens for eggs, and meat from animals such as chickens, goats or ducks. The predominant observed food source was the retail food environment. These outlets included grocery stores & supermarkets (eight families), convenience stores (six families), *tienditas en casa* (small neighborhood stores in homes) (two families), *pulgas* (flea markets) (two families), mobile fruit and vegetable vendors (one family), dollar stores (two families), mobile food vendors (three families), *carnicerias* (one family), bakeries (two families), fruit stands (four families), and *tortillerias* (one family). Food resources were accessed by walking to *tienditas* and convenience stores by three of the women, two entirely without vehicles and another without a vehicle during the week. They also asked friends and relatives to bring them items from grocery and other food stores. Households also relied on curbside service from mobile vendors of prepared foods and fruits and vegetables. Automobiles were used for more distant locations. Automobile use was common, although limitations in household resources were apparent in the condition of these automobiles. Many automobiles were in various states of disrepair, and residents often relied on friends and family for transportation. During the observational period, men performed all shopping, although all the women indicated they were regularly involved in shopping for food. The residents comfort with driving shaped this division of labor. One woman did not know how to drive and usually walked to the fruit stand and convenience stores. Her husband complemented this by shopping in his car.

*Storage* was performed entirely by women. Once food entered the home, it passed into the feminine domestic sphere. Men returned with groceries, but they were delivered to women for storage or immediate preparation. Storage categories were cold, dry, secure, and frozen. The levels of material hardship, all of which inspired creative in-home adaptations, framed the understanding of the use of items within the home for storage. These included limited financial resources, limited linkages to neighborhood infrastructure such as water and gas, limited space, and the permeability of the home to vermin, heat, cold, wind and rain.

Common items used for food and beverage storage in *colonia* homes included refrigerators, freezers, shelving and cabinets, plastic jugs, and kitchen counters. The limited resources of the owners often prohibited their acquisition of specialty storage items. In response, all of the families improvised storage solutions.

**Figure 2:**
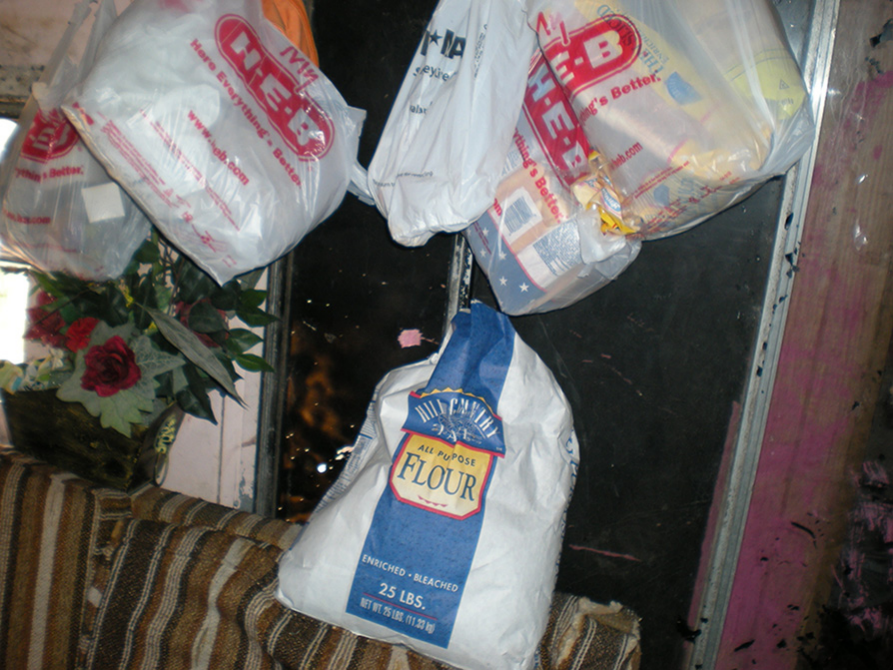
**Storage.** Dried goods in plastic bags and bag of flour are suspended from the wall as a form of storage.

**Figure 3:**
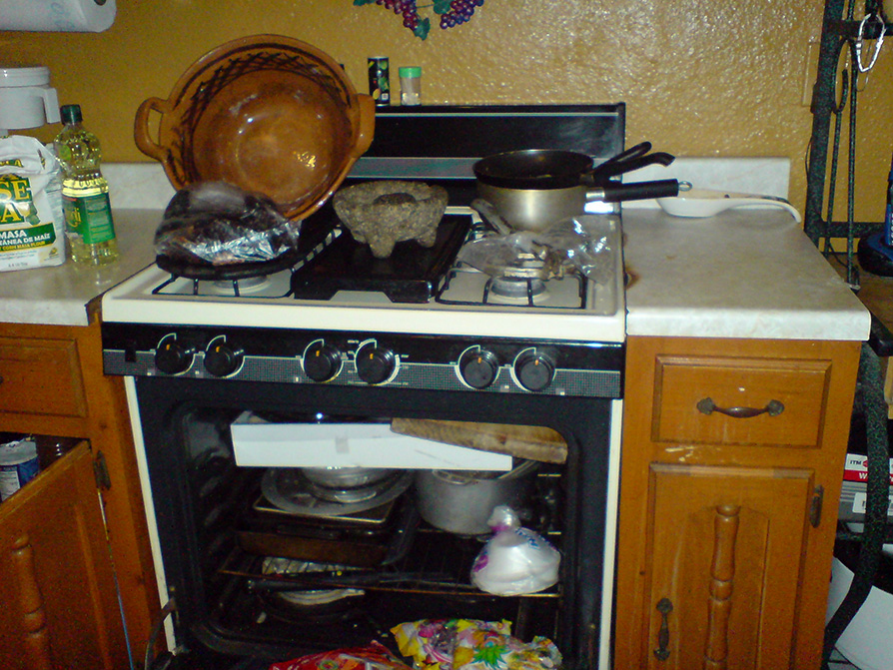
**Colonia household oven.** In the center on the top of the oven is a *molcajete* (mortar and pestle) resting on top of a *comal* (griddle). To the left are another *comal*, a bag of chiles, and an *olla* (clay pot for cooking beans). To the right are pots, and a tortilla press. Inside the oven are stored a wooden board configured for use as a cutting board, numerous pots and pans, another *comal*, and bags of candy.

Two families used broken refrigerators for dry storage. Plastic storage bags hung from walls were used for permanent storage of dried goods by three families (see Figure 2) and by one family to store waste. Microwaves (two families) and ovens (two families) were used to store dried goods (see Figure 3), and three families used oven-units to store pots and pans (see Figure 3).

The condition of the built home environment also influenced available space. The limited space in self-help housing, especially in the early stages of construction, called for the maximization of available space, regardless of presuppositions about the proper use of such spaces. For example, three families placed their refrigerators, functioning or not, outside the kitchen in living spaces or outdoors.

Another characteristic of the built home environment is the permeability of the home to vermin such as rats and insects. Management of vermin can be seen in the attempts to secure the storage of dried goods. Sufficient resources were not always present to acquire storage items specifically designed for protection from vermin. Some households solved this problem by adapting household appliances with secure seals for dry storage. One household initially lived in a small trailer that was transformed into a kitchen following the later addition of bedrooms and a living room. In the updated home, the residents installed a refrigerator in the living room and transformed the trailer’s built-in refrigerator into a location for sealed dry storage of medications, herbs and spices, and other dried goods. In two households, bread, tortillas, and other dried goods were stored in microwaves. Residents removed these items to use their microwave for heating and cooking. Following this standard use they returned the items to the microwave to protect them from rodents and insects.

Neighborhood infrastructure impacted water consumption. All of the study homes had running water. The authors have no information on whether or not this water was safe to drink, although a number of residents clearly had strong preferences against drinking tap water, a preference shared with many residents of South Texas *colonias*[14]. Five of the households used a variety of containers to store drinking water obtained from a *maquina de agua*; a dispenser of filtered water found at local grocery stores or in standalone facilities (kiosks), specifically for drinking water. This water was also used for cooking by three of the families.

*Preparation* was largely the task of women, and a clear public/private or outside/inside task division was observed. Women prepared food within the home for family and guests. In four of the families, men were either observed or mentioned by women as preparing food, but only outside for barbecues where friends and family were served. One woman joked about how she tried to persuade her husband to help out around the kitchen to no avail; another described her husband’s rare offers of assistance as ineffective, and another spoke of her husband’s helpfulness cleaning around the house while she was nursing their newborn child.

The use of tools in food preparation was largely the work of women. The use of kitchen tools was shaped by the three levels of material hardship, especially the limited resources available to obtain specialized tools. Also of importance were physical constraints such as the effects of inclement weather, and the preferences for specialized items designed to implement cultural repertoires such as the preparation of tortillas or other elements of Mexican cuisine.

An extensive range of food preparation practices was observed (see Table 2). These included frying and sautéing, simmering, steaming, defrosting, reheating, chopping, pureeing, mixing, grinding, soaking, grilling, roasting, cooking and pressing tortillas, toasting, coffee making, measuring, opening of cans and packages, cutting, juicing, cleaning vegetables, straining, dispensing water, warming, and degreasing. Limited resources did not prevent many households from obtaining items common to Mexican-American kitchens such as *molcajetes* (mortar and pestle) (see Figure 3) (five families); a range of items used to make tortillas such as *comals* (griddles) (five families) (see Figure 3), and electric (one family) and hand powered presses (four families) (see Figure 3); and blenders (four families) used to make salsas and to mix pancake batter and juices from frozen concentrate. With these exceptions, these were inexpensive items commonly available in the majority of Anglo-American households. There were few specialized items suitable for the preparation of one item. These included manual juicers, various tortilla presses, and toasters. Specialized items suitable for one task but usually employed in the making of multiple dishes included cutting boards and manual can openers. The majority of items were multipurpose.

**Table 2:** Methods of food preparation matched with specific tool (families numbered 1-8)

	**Method of food preparation**				
	**Frying/Sautéing**	**Boiling/Simmering**	**Steaming**	**Chopping/cutting/peeling**	**Mixing**
**Tool**	4-burner oven unit-burners (1-3, 6-7)	4-burner oven unit-burners (1-3, 6-7)	4-burner oven unit-burners (2-3)	Knives (1-8)	Blender (2)
	Portable stove top (4, 8)	Portable stove top (4-5, 8)	Aluminum foil (3)	Cutting board (1-4)	Mixing bowls (3)
	Skillet (cast iron and others) (1-8)	Portable pot and gasburner combo (1)	pots and pans (2, 3)	Plates (6, 7-8)	Baby bottle (8)
	Electric Skillet (3, 7-8)	Charcoal/wood Grill(2, 4, 7-8)		Plastic container top (5)	
	Griddle/comal (2, 7)	Pots and pans (1-8)		old lumber (8)	
		Ceramic bean pot(*olla*) (4)			
		Crock pot (4)			
	**Method of food preparation**				
	**Defrosting**	**Re-heating**	**Pureeing**	**Grinding**	**Soaking**
**Tool**	microwave (1-2, 5)	microwave (2-5)	Blender (1-5, 8)	Molcajete (1, 3, 8)	Bowl (2)
	**Method of food preparation**				
	**Juicing**	**Grilling**	**Opening cans**	**Toasting**	**Measuring**
**Tool**	Hand juicer (2)	Charcoal/wood grill(2, 3, 7)	Manual can opener(2, 3)	Toaster (2, 3)	Eyes and hands (1, 2, 3
			Knife (4, 6)		spoon (notmeasuring spoon) (1)
	**Method of food preparation**				
	**Coffee making**	**Pressing tortillas/gorditas**	**Cooking tortillas**	**Roasting**	**Warming water**
**Tool**	Stove-top pot withboiling water (1, 2	Tortilla press (3, 4)	Electric skillet (8)	Charcoal/wood grill (1)	Stove top (1, 2, 3, 6)
	Instant coffee (1, 2, 3, 8)	Electric tortilla press (8)		Gas burner (1)	Microwave (3)
	Coffee carafe (3)	Rolling pin (1, 3, 7)		Oven (7)	
	**Method of food preparation**				
	**Straining-Rinsing**	**Degreasing**			
**Tool**	Strainer (2, 3, 4)	Paper towels (2)			

Limitations in household resources were resolved through the use of commonly available tools instead of specialized items. The multiple uses of common household items are listed by family in Table 2. Two families employed a knife rather than a can opener. Three families used ceramic plates, pieces of lumber (see Figure 3), and plastic container tops as cutting boards. In many homes, cooks made-do with limited kitchen tools, often using the same item for multiple purposes. Measuring spoons and measuring cups were not observed in use in any of the homes. Instead, residents measured flour for tortillas with coffee cups, or simply with handfuls of flour and pinches of salt and baking powder. It was unclear whether this particular cultural repertoire was due to financial constraints which made the acquisition of precision measuring cups and spoons a luxury, the manner in which these women were trained to cook, or a combination of both factors.

Choice and use of stoves, ovens, and other cooking equipment was an aspect of food preparation that demonstrates the interlinking of socioeconomic, biological, and physical constraints within the household. Many distinct tools were used for cooking, including gas stove-and-oven units, one and two burner gas-units, electric stove-and-oven units, electric pans and griddles, microwaves, and wood and charcoal grills for outside cooking.

**Figure 4:**
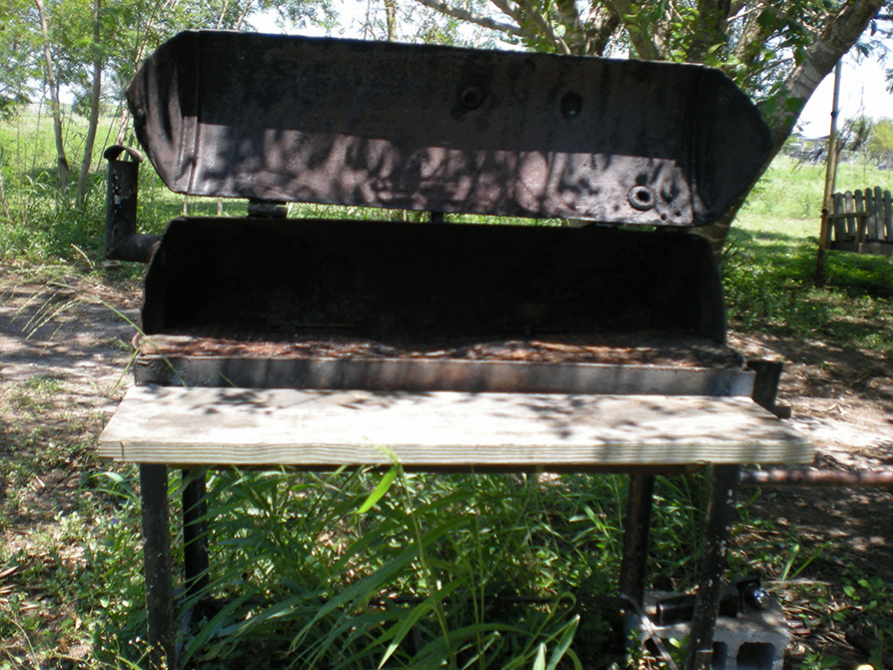
**Asador de patio (barbecue grill).** An *asador de patio* that has been repurposed from a hot-water heater.

Limitations in household resources were exhibited through the use of alternatives to larger and more expensive pieces of equipment such as stoves, and the use of less expensive and more flexible forms of fuel. Three families had broken stovetop and oven combination units which were repurposed as storage units. Replacements included less expensive units such as electric hot plates or a two-burner gas unit. When one family ran out of money to purchase propane for their burner they chose to cook beans outdoors over an open fire. While it was not clear if this was entirely a matter of conserving propane or electrical utilities or simply preference, all of the families either described or were observed cooking outside on a grill, often repurposed from an old butane tank or hot-water heater (see Figure 4), or on a *chiminea* (outdoor fireplace) using wood or charcoal. The food cooked on a grill might be meat, fish, or grilled vegetables, but also included items often cooked on a stovetop such as beans.

The built home environment also influenced the type of energy used to cook food. The trailers around which many of the homes were constructed provided a platform for one solution. The oven and stove combination units in these trailers were designed to use gas stored in canisters, often placed on the trailer exterior. This common element among self-help housing allows *colonia* residents to adapt to sparse or nonexistent neighborhood infrastructure. Many *colonias* are, at least initially, lacking in electrical or gas hookups. The use of gas canisters for fueling stoves and ovens was a simple and inexpensive solution to this lack of infrastructure, to homes that had not yet been connected to present infrastructure, or for households that were temporarily disconnected because of late payments.

The physical environment also impacted cooking practices. The majority of these homes were without central air-conditioning, central heating, or adequate insulation. On cold days, family members bundled up in warm clothing and spent as much time as possible under the covers in bed, or around the stove. Gas ovens, which were used nearly continuously on these cold days, achieved a dual purpose as cookers and heaters. If these observations were performed in the summertime, it is expected the ever present heat of South Texas would have inspired a different response, less use inside of ovens and other heat-producing cookers, and more cooking outdoors on grills and portable cookers.

These socioeconomic and gender based constraints, were demonstrated by the limited material resources of women and their primary location as homemakers. Given these constraints, women employed the resources at their disposal. Their cultural repertoires included an in-depth knowledge of the practical skills and ingredients of the Mexican home kitchen. Since they did not work outside the home, they devoted their time entirely to the practice of homemaking. The timesaving strategies employed by many modern households such as the use of specialized kitchen equipment, and a reliance on prepared food items, mixes and fast food were largely absent from these homes. In lieu of these innovations, women employed what Mammen and colleagues have referred to as human capital intensive techniques for coping with food insecurity [51], a flexible array of Mexican home cooking techniques and time intensive strategies in order to make do with limited material resources under conditions of material hardship.

*Feeding and Eating* were practices that involved eating utensils, and the tables, chairs, and other tools used to support food and the human body during the act of consumption. Many *colonia* residents used a conventional array of utensils including knives, forks, and spoons. One cultural repertoire was the frequent augmentation of utensil use with tortillas which served as both an inexpensive staple food item and utensil. All families ate corn tortillas, all but one family ate flour tortillas, and one family only used tortillas and a spoon in a common dish to eat their meals. All families with one exception ate tortillas with every meal. The common exceptions to tortilla consumption were snacks or breakfasts that consisted of *pan dulce* (sweet breads) or sandwiches.

Food consumption also entails the placement of food and the eater in convenient proximity. An eater dining at a table while seated at a chair is the normative case for many North Americans [52]. A number of eating arrangements were observed in *colonias*. The most common arrangement was a family seated in chairs at a table in the kitchen or living space. Other common arrangements included festive occasions such as family barbecues with meats and vegetables grilled outdoors. During these events, food would be consumed from a standing position around the grill. Indoor eating arrangements featured adaptations to limited space. One family in a home without room for an interior table and chair setting sat on a sofa and placed their food on their bed. Another family had room for a small chair and table setting with insufficient seating for the entire family. When this family dined together, many were seated on beds. Another family had a table and chairs in the living area adjoining the kitchen. This area was large enough for the children and the father to be seated at this table, but the mother and infant ate standing in the kitchen.

*Cleaning* is another category of practices constrained by the limited resources of the residents and the built home environment. Household cleaning was entirely performed by women. Cleaning was broken into three categories, personal hygiene, cleaning of food for preparation, and cleaning of the eating and food preparation areas. Brooms, mops, paper, sinks, paper towels, toothbrushes, and chemicals and soaps were all used for cleaning. Limited household resources inspired the adaptive use of a number of items. One home used a single functioning sink within the kitchen for cleaning vegetables and dishes, and for washing up after the bathroom and brushing teeth. Another used a piece of cardboard to clean instead of a dustpan. The limited availability of resources extended to the built environment. Few homes possessed a hot water heater, and stovetops were used to heat water to clean the home and for personal hygiene.

Management of food waste is another practice that demonstrates structural constraints on the use of household technology. Three categories were developed to describe food-waste management: temporary storage, cleaning, and disposal. Overlapping with the storage category, temporary storage of food waste was primarily shaped by two conditions, limited family resources to purchase items for temporary storage, and limited space. Many families used plastic grocery store sacks to store waste. Due to limited space, some families stored waste from the kitchen and from the bathroom or other rooms in one location.

Food-waste management was constrained by the lack of residential infrastructure and city services. Few residents had access to a trash pickup service. Some residents hauled their own trash to the city dump, but this was inconvenient and required a subscription to the city utility. Not all residents were able to provide this evidence, and not all had the time or the vehicle to deposit their own waste off site. These residents burned their waste.

Not all food waste was discarded. Some food waste was fed to pets or to chickens, ducks and goats. For example, one father and his children first shucked the *elotes* (corn) that he sold throughout the neighborhood in his family’s mobile food business and then fed the husks to their animals. The father observed that his children did not like to eat their own animals, but they were comfortable with eating animals and eggs raised by their neighbors. This process is indicative of the flow of resources through the community, a process of reciprocal exchange with neighboring families that was undoubtedly present but only indirectly observed in this study.

### Implications

*Colonias* are archetypal examples of the new-destination Mexican immigrant communities now broadly dispersed throughout the continental U.S. [6-10]. The importance of such food-preparation practices among women *colonia* residents is likely shared among women in other new destinations for Mexican immigrants, which bolsters the relevance of these findings.

The collection of detail on technological and other material household constraints is of importance when considering how household members cope with food insecurity. Material hardship is a useful conceptual linkage between food insecurity and other household demands including the material needs identified in this study. The satisfaction of demands for a secure food supply, food-related technology, medical care and insurance, clothing and other material necessities requires the application of economic or alternative resources. In impoverished households these demands may outstrip available resources and families may be forced to choose between material necessities. Thus, the concept of material hardship which has taken a prominent role in the sociological and demographic literature on poverty [34,53,54] should take on greater importance among public health practitioners and researchers for understanding the impact of household demands on the food security status of impoverished households [55].

Observations of eating arrangements that used beds and sofas for seating and as tables resembled arrangements described in limited-space low-income dwellings in British households by Charles and Kerr [29] and Mexican households by Lewis [56]. The findings from this study suggest future work on commensality and diet should consider eating arrangements. Participation in family meals and the family meal ritual have been associated with positive nutritional outcomes [57,58], and the spatial arrangement of meals and dining surfaces also influences dietary intake [52,59]. Future research on food insecure households should examine the impact of crowding and limited seating on commensality. It should also analyze the influence of household eating arrangements on food consumption to develop guidelines for improving eating arrangements in limited-resource households.

A number of practical considerations can be derived from examining food-related technological practices in the home food environment. Acknowledging these practices may impact nutritional interventions. For example, eating with a tortilla or a spoon implies a technological distinction that will likely influence caloric intake. Researchers that wish to capture and positively influence the consumption patterns of Mexican-immigrant communities must come to some understanding of what tortillas mean as a utensil for overall consumption patterns.

### Strengths

One of this paper’s strengths is the exploration of strategies for coping with material hardship as cultural repertoires. By identifying the ingredients, dishes and technological practices employed by these *colonia* residents to cope with material hardship, this paper adds to the recent conversation among cultural sociologists of how cultural repertoires are shaped by social and material circumstances [25-27,32]. It adds to this discussion by identifying socioeconomic constraints on the implementation of repertoires, and by rooting constraints in the biological and physical environment of South Texas *colonias*.

This study also possessed methodological strengths including the use of participant observation [38], a novel methodology in this setting. Participant observation facilitated: 1) scholarly access to observational data on day-to-day food related activities and 2) a description of the flow of food stuffs and food-related practices within households [15,50], including constraints on the availability of food, household technology, and other material goods, and the creative practices and adaptations employed to resolve these limitations. Another methodological strength is the use of community based *promotora-*researchers who served as cultural brokers and lessened the distance between university based researchers and community members. The *promotora-*researchers collected high quality data and provided intimate observations of the households, permitting access to otherwise inaccessible day-to-day household activities.

### Limitations

This research was not without limitations. The observations were performed in the winter. Observations collected across multiple seasons would have allowed a broader categorization of the factors that impacted food-related technological behaviors. The sample also was of a small number of households in one region of South Texas and conducted over a single month, and can thus not be described as capturing the entire breadth of food-related technological practices or forms of material hardship to be seen among South Texas *colonia* households.

## Conclusions

The *colonia* households in this study evidenced forms of material hardship including limited space in their homes; poor health conditions; limited insurance and health care access; inadequate housing, and food insecurity. To adapt to these conditions, women *colonia* residents creatively employed cultural repertoires including the dishes and inexpensive staple ingredients of regional cuisine, and their limited and multipurpose forms of kitchen technology. In *colonia* households, women expressed a creative capacity to acquire, store, prepare, feed and eat, clean and dispose of food waste that was nevertheless structured by the constraints endemic to their status as women Mexican-immigrants residing in households/homes and neighborhoods constrained by material hardship.

## Competing interests

The authors declare that they have no competing interests.

## Authors’ contributions

JRS conceived and designed the study. WRD analyzed data. WRD, JRS, CMJ and JSJ contributed to data interpretation. WRD prepared the first draft. WRD, JRS, and CMJ made revisions, JSJ supervised and conducted data collection, and all authors read and approved the final manuscript.

## References

[B1] Sharkey JR Horel S Han DK Huber JC Association between neighborhood need and spatial access to food stores and fast food restaurants in neighborhoods of Colonias Int J Health Geogr 2009 8 9 10.1186/1476-072X-8-9 19220879 PMC2653484

[B2] Sharkey JR Dean WR Johnson CM Association of household and community characteristics with adult and child food insecurity among Mexican-origin households in colonias along the Texas-Mexico border Int J Equity Health 2011 10 19 10.1186/1475-9276-10-19 21569496 PMC3118344

[B3] Mier N Ory MG Zhan D Conkling M Sharkey JR Burdine JN Health-related quality of life among Mexican Americans living in colonias at the Texas-Mexico border Soc Sci Med 2008 66 1760 1771 10.1016/j.socscimed.2007.12.017 18261832

[B4] Institute for Health Promotion Research: UT Health Science Center, San Antonio South Texas Health Status Review South Texas Health Status Review 2010 Institute for Health Promotion Research: UT Health Science Center, San Antonio, San Antonio

[B5] St. John JA Johnson CM Dean WR Arandia G Empowerment: evolution of promotoras as promotora-researchers in the Comidas Saludables & Gente Sana en las Colonias del Sur de Tejas (Healthy Food and Healthy People in South Texas Colonias) Program. J Prim Prev10.1007/s10935-013-0296-1 23404423

[B6] Ward PM Colonias and Public Policy in Texas and Mexico: Urbanization by Stealth 1999 University of Texas Press, Austin, TX

[B7] Zúñiga V Hernández-León R New Destinations: Mexican Immigration in the United States 2006 Russell Sage, New York

[B8] Gray VB Cossman JS Dodson WL Byrd SH Dietary acculturation of Hispanic immigrants in Mississippi Salud Pub Mex 2005 47 351 360 10.1590/S0036-36342005000500005 16323528

[B9] Cornfield DB Ansley F, Shefner J Immigrant labor organizing in a "new destination city": approaches to the unionization of African, Asian, Latino, and Middle Eastern workers in Nashville In Global Connections & Local Receptions: New Latino Immigration to the Southeastern United States 2009 University of Tennessee Press, Knoxville, TN 279 297

[B10] Jensen L Duncan CM New Immigrant Settlements in Rural America: Problems, Prospects, and Policies 2010 3 Carsey Institute, University of New Hampshire, Durham, New Hampshire 1 34

[B11] Williams R The Colonia Initiatives Program of the Office of Texas Secretary of State Final Report in Response to Senate Bill 827 by Senator Judith Zaffirini and Representative Ryan Guillen, 79th Regular Session, Texas Legislature. Tracking the Progress of State-funded Projects that Benefit Colonias 2006 Office of Texas Secretary of State, Austin, TX 1 137

[B12] Fronczek P Income, Earnings, and Poverty from the 2004 American Community Survey 2005 Economic and Statistics Administration, US Census Bureau, Washington, DC

[B13] U.S. Census Bureau American Fact Finder 2010 U.S. Census Bureau, Washington D.C http://factfinder2.census.gov/faces/nav/jsf/pages/index.xhtml

[B14] Mroz RC Morales LL VanDerslice J Health and hygiene in the colonias: water and disease Fam Com Health 1996 19 49 58

[B15] Sobal J Kettel Khan L Bisogni C A conceptual model of the food and nutrition system Soc Sci Med 1998 47 853 863 10.1016/S0277-9536(98)00104-X 9722106

[B16] Silva E The cook, the cooker and the gendering of the kitchen Sociol Rev 2000 48 612 628 10.1111/1467-954X.00235

[B17] Silverstone R Hirsch E Morley D Information and communication technologies and the moral economy of the household Consuming technologies: Media and information in domestic spaces 1992 Routledge, London, U.K. 115 131

[B18] Loe M Doing it my way: old women, technology and well being Sociol Health Illn 2010 32 319 334 10.1111/j.1467-9566.2009.01220.x 20149150

[B19] Law J Law J, Hassard J After ANT: complexity, naming and topology In Actor network theory and after 1999 Blackwell, Oxford 1 14

[B20] Latour B Feenberg A A door must be either open or shut: A little philosophy of techniques Technology and the Politics of Knowledge 1995 Indiana University Press, Bloomington, Indiana 272 281

[B21] Davidson C A Woman's Work Is Never Done: A History of Housework in the British Isles, 1650-1950 1986 Chatto and Windus, London

[B22] Bell G Kaye J Designing Technology for Domestic Spaces: A Kitchen Manifesto Gastronomica 2002 2 46 62 10.1525/gfc.2002.2.2.46

[B23] Isenstadt S Visions of Plenty: Refrigerators in America around 1950 J Design Hist 1998 11 311 321 10.1093/jdh/11.4.311

[B24] Zavella P Reflections on diversity among Chicanas Frontiers: A Journal of Women Studies 1991 12 73 85 10.2307/3346849

[B25] Swidler A Culture in Action: Symbols in Strategies Am Sociol Rev 1986 51 273 286 10.2307/2095521

[B26] Small ML Harding D Lamont M Reconsidering culture and poverty Ann Am Acad Pol Soc Sci 2010 629 6 27 10.1177/0002716210362077

[B27] Lamont M Thévenot L Lamont M, Thévenot L Introduction: Toward a renewed comparative cultural sociology Rethinking comparative cultural sociology 2000 Cambridge University Press, Cambridge, U.K 1 24

[B28] Devine C A life course perspective: understanding food choices in time, social location, and history J Nutr Educ Behav 2005 37 121 128 10.1016/S1499-4046(06)60266-2 15904575

[B29] Charles N Kerr M Women, food, and families 1988 Manchester University Press, U.K.

[B30] Dean WR Sharkey JR Cosgriff-Hernández K-K Martinez AR Ribardo J Diaz-Puentes C I can say that we were healthy and unhealthy”: food choice and the reinvention of tradition Food, Culture and Society 2010 13 573 594

[B31] Sewell WH A theory of structure: Duality, agency, and transformation Am J Sociol 1992 98 1 29 10.1086/229967

[B32] Hannerz U Soulside: Inquiries into Ghetto Culture and Community 1969 Columbia University Press, New York

[B33] Sobal J Bisogni C Devine C Jastran M Shepherd R, Raats M A conceptual model of food choice process over the life course In The psychology of food choice 2006 CABI International, Oxfordshire, U.K 1 18

[B34] Beverly S Measures of material hardship J Poverty 2001 5 23 41 10.1300/J134v05n01_02

[B35] Dasgupta P An inquiry into well-being and destitution 1995 Oxford University Press, USA

[B36] Collins HM The TEA set: tacit knowledge and scientific networks Sci Stud 1974 4 165 185 10.1177/030631277400400203

[B37] Collins H Schatzki TR, Knorr-Cetina K, Savigny E What is tacit knowledge In The practice turn in contemporary theory 2001 Routledge, London 107 119

[B38] Jorgensen DL Participant observation: A methodology for human studies 1989 Sage Publications, Inc, Thousand Oaks, CA

[B39] Sukovic M Sharf BF Sharkey JR John JS Seasoning for the Soul: Empowerment Through Food Preparation Among Mexican Women in the Texas Colonias Food and Foodways 2011 19 228 247 10.1080/07409710.2011.600126

[B40] Hamelin AM Habicht JP Beaudry M Food insecurity: consequences for the household and broader social implications J Nutrit 1999 129 525 10.1093/jn/129.2.525S 10064323

[B41] Frongillo EA Rauschenbach BS Olson CM Kendall A Colmenares AG Questionnaire-based measures are valid for the identification of rural households with hunger and food insecurity J Nutrit 1997 127 699 9164989 10.1093/jn/127.5.699

[B42] Long-Solís J Vargas LA Food culture in Mexico 2005 Greenwood Press, Westport, CT

[B43] de la Peña Brown MH Una Tamalada: The Special Event West Folk 1981 40 64 71 10.2307/1499850

[B44] Williams B Brown LK, Mussell K Why Migrant Women Feed Their Husbands Tamales: Foodways as a basis for a Revisionist View of Tejano Family Life In Ethnic and Regional Foodways in the United States 1984 University of Tennessee Press, Knoxville

[B45] Abarca M Voices in the Kitchen: Views of Food and the World from Working-Class Mexican and Mexican American Women 2006 Texas A&M University Press, College Station, TX

[B46] Pilcher JM Que vivan los tamales!: Food and the Making of Mexican Identity 1998 University of New Mexico Press, Albuquerque

[B47] Brandes S Ritual eating and drinking in Tzintzuntzan: A contribution to the study of Mexican foodways West Folk 1990 49 163 175 10.2307/1499404

[B48] Johnson C Sharkey J Dean W It's all about the children: a participant-driven photo-elicitation study of Mexican-origin mothers' food choices BMC Women's Health 2011 11 1 15 10.1186/1472-6874-11-1 21943081 PMC3200150

[B49] Pinto K Coltrane S Divisions of Labor in Mexican Origin and Anglo Families: Structure and Culture Sex Roles 2009 60 482 495 10.1007/s11199-008-9549-5

[B50] DeVault M Feeding the Family: The Social Organization of Caring as Gendered Work 1994 University of Chicago Press, Chicago

[B51] Mammen S Bauer J Richards L Understanding persistent food insecurity: A paradox of place and circumstance Soc Indic Res 2009 92 151 168 10.1007/s11205-008-9294-8

[B52] Sobal J Wansink B Kitchenscapes, tablescapes, platescapes, and foodscapes: Influences of microscale built environments on food intake Environ Behav 2007 39 124 10.1177/0013916506295574

[B53] Beverly S Material hardship in the United States: Evidence from the survey of income and program participation Soc Work Res 2001 25 143 151 10.1093/swr/25.3.143

[B54] Mayer S Jencks C Poverty and the distribution of material hardship J Hum Res 1989 24 88 114 10.2307/145934

[B55] Johnson CM Sharkey JR Dean WR Indicators of Material Hardship and Depressive Symptoms Among Homebound Older Adults Living in North Carolina J Nutr Gerontol Geriatr 2011 30 154 168 10.1080/21551197.2011.566527 21598164

[B56] Lewis O The possessions of the poor Sci Am 1969 221 114 124 10.1038/scientificamerican1069-114 5809583

[B57] McIntosh W Dean W Torres C Anding J Kubena K Nayga R Meiselman HL The American Family Meal In Meals in Science and Practice: Interdisciplinary Research and Business Applications 2009 Woodhead Publishing Limited, Cambridge, U.K 190 235

[B58] McIntosh WA Kubena KS Tolle G Dean WR Jan J Anding J Mothers and Meals: The Effects of Mothers' Meal Planning and Shopping Motivations on Children's Participation in Family Meals Appetite 2010 55 623 628 10.1016/j.appet.2010.09.016 20870001

[B59] Sobal J Wansink B Blass EM Built Environments and Obesity Obesity: Causes, Mechanisms, Prevention, and Treatment 2008 Sinauer Associates, Sunderland, MA 281 299

